# Personalized Treatment Planning Automation in Prostate Cancer Radiation Oncology: A Comprehensive Dosimetric Study

**DOI:** 10.3389/fonc.2021.636529

**Published:** 2021-06-01

**Authors:** Savino Cilla, Carmela Romano, Vittoria E. Morabito, Gabriella Macchia, Milly Buwenge, Nicola Dinapoli, Luca Indovina, Lidia Strigari, Alessio G. Morganti, Vincenzo Valentini, Francesco Deodato

**Affiliations:** ^1^ Medical Physics Unit, Gemelli Molise Hospital–Università Cattolica del Sacro Cuore, Campobasso, Italy; ^2^ Radiation Oncology Unit, Gemelli Molise Hospital–Università Cattolica del Sacro Cuore, Campobasso, Italy; ^3^ Radiation Oncology, IRCCS Azienda Ospedaliero–Universitaria di Bologna, Bologna, Italy; ^4^ DIMES, Alma Mater Studiorum Bologna University, Bologna, Italy; ^5^ Radiation Oncology Department, Fondazione Policlinico Universitario A. Gemelli–Università Cattolica del Sacro Cuore, Rome, Italy; ^6^ Medical Physics Unit, Fondazione Policlinico Universitario A. Gemelli–Università Cattolica del Sacro Cuore, Rome, Italy; ^7^ Medical Physics Unit, IRCCS Azienda Ospedaliero–Universitaria di Bologna, Bologna, Italy; ^8^ Istituto di Radiologia, Università Cattolica del Sacro Cuore, Rome, Italy

**Keywords:** automated planning, personalized, prostate cancer, VMAT (volumetric modulated arc therapy), pinnacle, dosimetric analysis

## Abstract

**Background:**

In radiation oncology, automation of treatment planning has reported the potential to improve plan quality and increase planning efficiency. We performed a comprehensive dosimetric evaluation of the new Personalized algorithm implemented in Pinnacle^3^ for full planning automation of VMAT prostate cancer treatments.

**Material and Methods:**

Thirteen low-risk prostate (without lymph-nodes irradiation) and 13 high-risk prostate (with lymph-nodes irradiation) treatments were retrospectively taken from our clinical database and re-optimized using two different automated engines implemented in the Pinnacle treatment system. These two automated engines, the currently used Autoplanning and the new Personalized are both template-based algorithms that use a wish-list to formulate the planning goals and an iterative approach able to mimic the planning procedure usually adopted by experienced planners. In addition, the new Personalized module integrates a new engine, the Feasibility module, able to generate an “*a priori*” DVH prediction of the achievability of planning goals. Comparison between clinically accepted manually generated (MP) and automated plans generated with both Autoplanning (AP) and Personalized engines (Pers) were performed using dose-volume histogram metrics and conformity indexes. Three different normal tissue complication probabilities (NTCPs) models were used for rectal toxicity evaluation. The planning efficiency and the accuracy of dose delivery were assessed for all plans.

**Results:**

For similar targets coverage, Pers plans reported a significant increase of dose conformity and less irradiation of healthy tissue, with significant dose reduction for rectum, bladder, and femurs. On average, Pers plans decreased rectal mean dose by 11.3 and 8.3 Gy for low-risk and high-risk cohorts, respectively. Similarly, the Pers plans decreased the bladder mean doses by 7.3 and 7.6 Gy for low-risk and high-risk cohorts, respectively. The integral dose was reduced by 11–16% with respect to MP plans. Overall planning times were dramatically reduced to about 7 and 15 min for Pers plans. Despite the increased complexity, all plans passed the 3%/2 mm γ-analysis for dose verification.

**Conclusions:**

The Personalized engine provided an overall increase of plan quality, in terms of dose conformity and sparing of normal tissues for prostate cancer patients. The Feasibility “*a priori*” DVH prediction module provided OARs dose sparing well beyond the clinical objectives. The new Pinnacle Personalized algorithms outperformed the currently used Autoplanning ones as solution for treatment planning automation.

## Introduction

In radiation oncology, the quality of treatment planning has a major impact on clinical outcomes as well demonstrated in several clinical trials (1, 2). Despite the worldwide implementation of the ICRU83 guidelines (3), local treatment planning protocols still have a major impact on plan quality. A recent multicentric study (4) reported that the adherence to ICRU83 recommendations for dose prescription were relatively poor, with statistically significant variability in target dose coverage and dose homogeneity among institutions. The relationship between the plan quality and the clinical outcomes has been recently reported, proving that failures to adhere to protocol guidelines are associated with reduced local control and survival and potentially increased toxicity (5). In particular, in prostate radiotherapy, the cost was found particularly high. An analysis of frequency and clinical severity of quality deficiencies in planning on the RTOG126 protocol demonstrated the critical impact of suboptimal plans on rectal complications (6). Of the 219 enrolled patients, 42.9 and 9.1% had a ≥5% a ≥10% excess risk rectal complications, and re-planning reported significant NTCP reductions while maintaining optimal target coverage. The observed toxicities were consistent with the current radiobiological modeling.

With the advent of intensity-modulated techniques (IMRT), also in the form of rotational volumetric modulated arc therapy (VMAT), radiation treatment plans have become increasingly complex. The conventional treatment planning for these techniques requires many manual processes. Many compromises must be uniquely negotiated for each patient because the optimal dose distribution maximizing the therapeutic ratio for a given patient is never known *a priori* and the only chance to planners is to manage many competing parameters in a trial-and-error process. The clinical and dosimetric objective are then iteratively adjusted during several optimization processes in order to generate clinically acceptable treatment plans. This procedure is not only time-consuming but could also influence the consistency and plan quality, inherently dependent on the individual skill of the planner (7, 8).

In the last years, the applications of artificial intelligence in radiation oncology translated in many technological advancements, including patient outcomes modeling, organs auto-segmentation, dose prediction, and treatment plan automation (9). Different approaches have been proposed so far for the automation of treatment planning including knowledge-based optimization, multi-criteria optimization, and template-based strategies. Knowledge-based (10, 11) concepts rely on predictive models built on statistical analysis of a large number of previous plans, providing an estimate of the dose distribution and dose-volume histograms (DVH) for any new patient. This approach has been implemented in the RapidPlan engine, commercially implemented in the Varian Eclipse treatment planning system (TPS) (Varian Medical Systems, Palo Alto, USA), reporting a general improvement in the inter-consistency of treatment plans (12–15). The multi-criteria optimization approach (16) is based on the generation of the so-called “Pareto-optimal” treatment plans (i.e. plans for which improving one criterion value is not possible unless some other criterion value deteriorates), allowing the user to navigate interactively through these solutions in order to obtain one that yields the desired trade-off between different criteria. This strategy has been implemented in the RayStation TPS (Raysearch, Stockholm, Sweden) (17) and in the Erasmus-Icycle algorithm developed at Erasmus MC-Cancer Institute in Rotterdam (18–20). In particular, the Raystation TPS provides a pool of output plans on the Pareto-optimal surface, leaving the user to define the best final plan, while the Erasmus-iCycle engine supplies the most Pareto-optimal plan according to a clinical wish-list of dosimetric objectives. The template-based approach has been implemented in the Pinnacle TPS (Philips Medical Systems, Fitchburg, WI, USA) in the so-called Autoplanning engine (21). In this strategy, the planning optimization process uses a template to formulate all the planning goals and an iterative approach able to mimic the planning procedure usually adopted by experienced planners to generated high-quality plans. This approach has been investigated in several publications for prostate (21–23), head-neck (24), and for extracranial stereotactic treatments (25, 26) reporting an overall increase of plan quality together with a substantial reduction of planning time and inter-planner variations.

A new generation of advanced optimization algorithms for inverse planning, called Personalized planning, is under current investigation in the new Pinnacle Evolution TPS. Pinnacle Personalized is an advanced replacement of the optimizer used in Autoplanning, aiming to further improve the overall plan quality and the speed of IMRT and VMAT automated optimization. In particular, this new engine presents an advanced technology called Feasibility which allows an estimation of the best possible sparing of the OARs in order to inform the planner “*a priori*” about the achievability of treatment planning goals (27). Assuming a complete target dose coverage and an ideal fall-off from the prescription doses at the targets boundary, a feasibility dose-volume histogram (fDVH) can be calculated in less than 1 min before the start of optimization process. This fDVH divides the dose space into regions that are impossible, difficult, challenging, or probable for each OAR. This *a-priori* knowledge allows the planner to personalize the planning goals for all OARs according to each patient geometry, in addition to the initial objectives defined in the treatment template.

To the best of our knowledge, no previous studies have investigated the potential of Pinnacle Personalized for automation of planning process in prostate cases.

This study aimed to provide a comprehensive dosimetric evaluation of Pinnacle Personalized potential for the radiotherapy of prostate cancer in the two scenarios of low-risk and high-risk prostate cancer. In the last case, the large irregular-shaped targets volumes, the simultaneous multiple dose prescriptions, and the several organs-at-risk (OARs) adjacent to the targets represent a major challenge for the generation of high-quality plans. In this paper, we then hypothesized the potential of these new automated planning algorithms to improve consistency and plan quality and we discussed how the introduction of treatment planning automation affected the workflow in the clinical practice.

## Material and Methods

### Patient Selection, Simulation, Volume Definition, and Dose Prescriptions

This retrospective planning study included patients previously treated at our institution for prostate cancer with VMAT technique. Twenty-six patients were included, 13 consecutive patients in each of the following two categories: a) low-risk prostate and b) high-risk prostate.

All patients underwent a CT-simulation (3 mm slice thickness) in a vacuum-lock device in a supine position, with specific instructions to empty the bladder and rectum before the simulation and each treatment fraction. The following structures were contoured: prostate, regional lymph-nodes, the entire bladder, the rectum (from ischium to sigmoid flexure), the small bowel, and the femoral heads. All targets and normal tissues were segmented and delineated by a radiation oncologist and then reviewed by a senior radiation oncologist with more than 10 years experience (FD).

Group (a): Low-risk prostate cases. The clinical target volume (CTV65) included the entire prostate and the caudal 2 cm of the seminal vesicles. The planning target volume (PTV65) was defined by adding a margin of 6 mm in the posterior direction and 8 mm in all other directions. Dose prescription for PTV65 was 65 Gy in 25 fractions.

Group (b): High-risk prostate cases. The clinical target volume 1 (CTV65) was defined as the prostate plus the seminal vesicles. The CTV45 included the obturator, internal and external iliac, and presacral lymph nodes. The two planning target volumes, PTV65 and the PTV45, were defined by adding 8-mm margins (6 mm posteriorly) to the CTV65 and 8-mm margins to the CTV45, respectively. High-risk prostate cases were planned using a simultaneous integrated boost (SIB) scheme derived from the literature, calculated based on the biologically equivalent dose (BED) for acute toxicity and tumor response. The regimen consisted of 65 and 45 Gy simultaneously delivered to the prostate and to the lymph-nodal volumes in 25 fractions.

In both scenarios, this fractionation translates to the equivalent delivery of 76.1 Gy in a standard 2 Gy/fraction (EQD2) (using αβ = 1.5) to the prostate. For the OARs, this scheme produced an EQD2 dose of 72.8 Gy (using αβ = 3). The fractionation scheme was designed to obtain a high biochemical control while maintaining a low OAR toxicity profile.

Planning objectives for the targets and organs-at-risk are reported in **Table 1**. The treatment goal was to deliver more than 95% of the prescribed dose to more than 98% of each PTV (D98% ≥ 95%) and less than 105% of prescribed doses to 2% of PTVs (D2% ≤ 105%). D98% and D2% represent the doses to 2 and 98% of the PTVs and are defined as metrics for near-minimum and near-maximum doses, respectively. Tolerance doses to the rectum, bladder, femurs, and small bowel were obtained from the Quantec guidelines (28). The Quantec doses were converted to their radiobiological equivalents (using BED and αβ = 3 Gy) to determine the tolerances listed in **Table 1**.

**Table 1:** Clinical objectives for treatment planning. For the OARs, the Quantec doses were converted to their radiobiological equivalents (using BED and αβ = 3 Gy) to determine the corresponding dose-volume objectives in the present hypofractionated regimen.

	Dose (cGy)		Volume
Low-risk prostate cases			
PTV6500	6,175		≥98%
	6,370		≥95%
	6,825		<2%
High-risk prostate cases			
PTV6500	6,175		≥98%
	6,370		≥95%
	6,825		<2%
PTV4500	4,275		≥98%
	4,410		≥95%
	4,725		<2%
	Dose (Quantec cGy)	Dose (Eq cGy)	Volume
Organs-at-risk			
Rectum	5,000	5,000	<50%
	6,000	5,690	<35%
	6,500	6,010	<25%
	7,000	6,330	<20%
Bladder	6,500	6,010	<50%
	7,000	6,330	<35%
Small bowel	1,500	1,500	<120 cc
Femoral heads	4,500	4,500	<2%

### Treatment Planning

For each patient, the clinically manual VMAT plan, generated by an experienced medical physicist according to local written protocols, was used as the reference plan. Automated VMAT plans were generated with both Pinnacle Autoplanning and Pinnacle Personalized modules, and compared with the clinically accepted ones. More details will be provided in the next paragraph. All plans were generated for an Elekta VersaHD linac (Elekta Ltd., Crawley, UK). Dose calculations were performed using the collapsed cone convolution dose calculation algorithm with a 2-mm grid resolution.

### Manual VMAT Planning

Clinical manual VMAT plans (MP) were generated with one arc for low-risk prostate cases and with the “dual-arc” feature for the high-risk cases, using the inverse optimization process previously described in more details (29) for coplanar 6 MV photon beams. A full gantry rotation was described by a sequence of 90 control points, i.e. one every 4°. Collimator was set at 10° to minimize the tongue-and-groove cumulative effect. All plans were optimized by a medical physicist with 10 years’ experience in VMAT planning, with the aim to obtain the highest quality plans and a reduction of inter-planner variability. MP plans were those clinically used for patient treatment; no manual plan was regenerated. All manual plans were optimized without time pressure and limitations. In addition, MP plans underwent a clinical judgment before their acceptability for delivery by two radiation oncologists and a medical physicist, following strict in-house implemented quality assurance procedures (30).

### Automated VMAT Planning With Pinnacle Autoplanning

AP plans were created using the Autoplanning module implemented in the Pinnacle^3^ Version 16.0 (Philips Medical Systems, Fitchburg, WI, USA), designed to automate the inverse planning optimization process by utilizing a so-called “Technique”, i.e. a template of parameters that can be customized for each treatment protocol and tumor site. The Autoplanning engine has been extensively described in a previous study (21). Briefly, the Technique includes the definition of all beam parameters, dose prescriptions, and planning objectives for PTVs and OARs and was defined on the same beam parameters, dose prescription, and clinical objectives adopted for the MP plans. The objectives for the two PTVs were only defined by numbers close to prescription doses (in our experience we chose as target goals the prescription doses plus 1 Gy, so as to avoid possible under dosage in PTVs boundary). The OARs objectives included maximum dose, mean dose, and dose-volume histogram points; they can have three different priority levels (high, medium, and low) and can be set compromised or uncompromised. Three parameters must be set: (a) the tuning balance (i.e. the balance between target dose conformity and OARs sparing), (b) the dose fall-off margin (i.e. the distance across which the dose should decrease from 80 to 20% in an automatically generated tuning ring structure around the PTVs), and (c) the Cold-Spot ROI (i.e. the identification of cold regions inside the PTVs and the automatic creation of new tuning volumes and relative dose objectives to increase dose in the last optimization loops).

At the start of the optimization, the Autoplanning module iteratively performs several optimization cycles in order to achieve the dosimetric objectives defined in the Technique. Specifically, the optimizer automatically generates various support structures in order to increase the dose conformity and to drive the OARs sparing as much as possible. These structures include (a) rings around the PTVs to control the dose fall-off, (b) residual target structures where overlaps between non-compromised OARs are removed, (c) residual OAR structures where overlaps between target are removed, (d) body structure used to control the dose spillage, and (e) internal target structures to control target dose homogeneity. During the optimization loops, extra objectives are automatically created for these new structures with the aim to continually spare the OARs at constant target dose coverage. All objective dose and weight parameters are tuned using proprietary algorithms.

### Automated VMAT Planning With Pinnacle Personalized

Pers plans (Pers) were optimized with the Personalized module implemented in the version 16.4.1 of Pinnacle^3^ Evolution TPS (Philips Medical Systems, Fitchburg, WI, USA). This module is an evolution of the currently used “Autoplanning” module. It combines new advanced Philips-proprietary optimization algorithms with the Feasibility engine, a new algorithm able to create personalized objectives for the OARs based on actual patient anatomy (31). In particular, the Personalized module features two powerful robust algorithms, the Limited memory Broyden-Fletcher-Goldfarb-Shanno (L-BFGS) for fluence map optimization and the Layered Graph for aperture size and shape optimization. The L-BFGS algorithm is used to reduce the dose grid matrix—which contains over a million discrete voxels and 100,000 different parameters—to a more workable size. From a matrix that contains ≥100 billion entries used to shape the dose distribution, L-BFGS creates considerably smaller matrices that yield roughly equivalent results as the larger matrix. This reduces the time needed for optimization by reducing the memory needs for computation and storage of entries. Then the Layered Graph algorithm is used to generate a finite number of MLC shapes in order to adhere to linac machine constraints for deliverability. As for the Autoplanning engine, a Technique was defined using the same beam parameters, dose prescription, and clinical objectives adopted for MP and AP plans.

Before the start of optimization process, planners can also create personalized objectives for the OARs based on actual patient anatomy. This task is performed by the Feasibility module, originally developed in the PlanIQ software (Sun Nuclear Corporation, Melbourne, FL, USA) and now integrated into the Pinnacle Personalized planning workflow. This module is a model-based calculation engine that uses the patient’s CT images, the prescription doses and the geometric relationship between the target volumes and OAR to create the so-called feasibility-DVH (fDVH) for each OAR. The mathematical description of the Feasibility calculation module has been thoroughly described by Ahmed et al. (27). Briefly, based on the calculation on a benchmark grid dose using energy-specific low-dose and high-dose kernels, the Feasibility module is able to generate the DVH “space” for a given OAR by computing a feasibility level (f) ranging between 0 (unachievable level) and 1 (easily achievable level). An iso-feasibility curve is then created by joining the points in the DVH with the same value of f. The DVH corresponding to f = 0 value is obtained assigning the full coverage to the target volumes and the minimum dose that any voxel outside targets could receive. This situation represents the “ideal” dose distribution and the relative DVH the best possible sparing curve (unachievable by design). Then, a feasibility level is calculated for every point above the f = 0 curve, considering the normalized distance of this point to the f = 0 curve; a closeness-to-feasibility function is used to convert this distance to a feasibility level (27). At the end of the calculation phase, a fDVH “space” for each OAR is generated and presented to the planner as a qualitatively picture divided in four main areas: a) an “unachievable” region (presented in red color) if full targets coverage is preserved, having the f = 0 curve as upper boundary, b) a “difficult” to achieve region (presented in orange color), which includes all DVH curves with f values ranging between 0 and 0.1, c) a “challenging” to achieve region (presented in yellow color) with curves ranging between f = 0.1 and f = 0.5, and d) the “easy” to achieve region from with curves ranging between f = 0.5 and f = 1.

**Figure 1:**
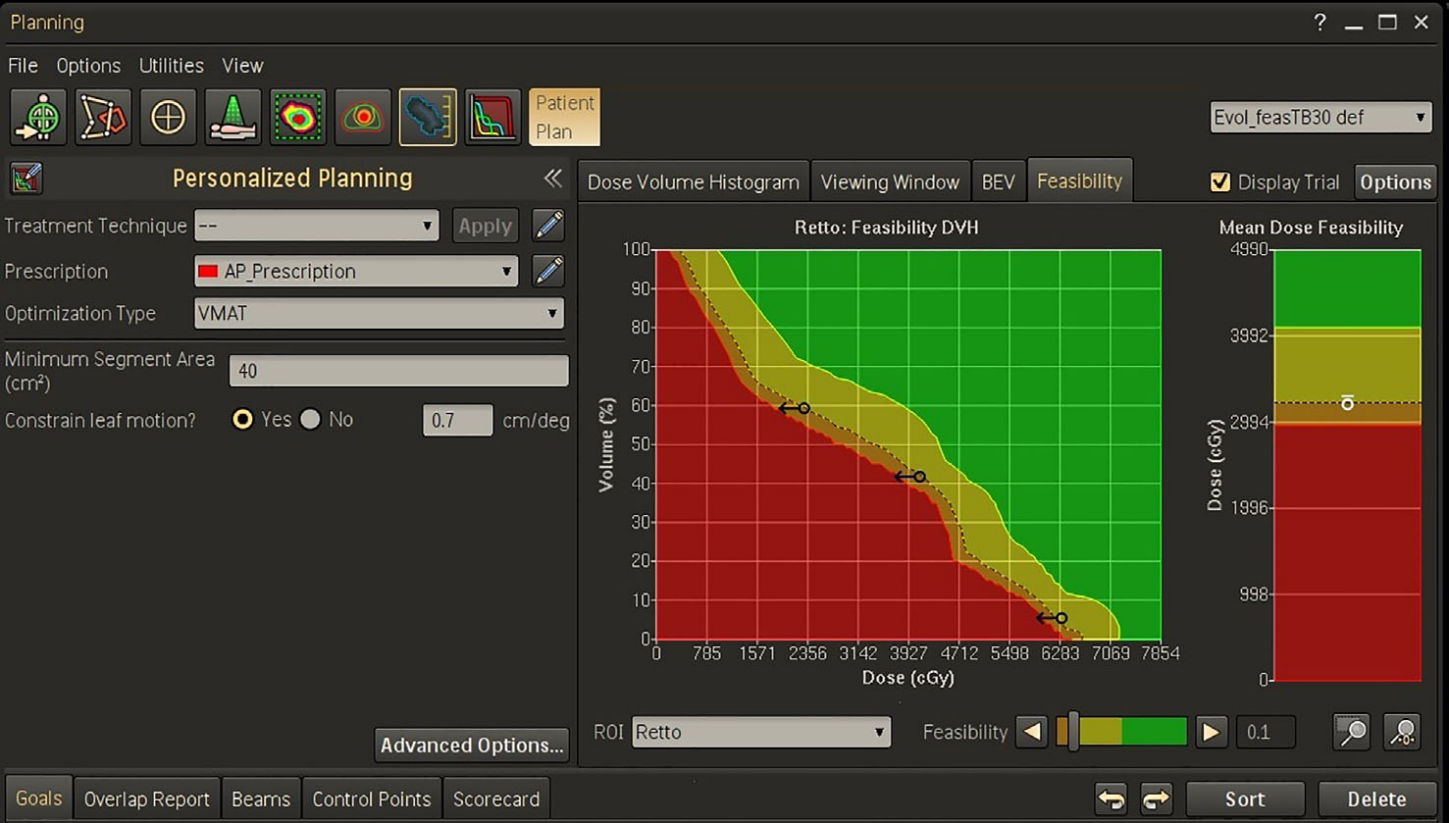
Feasibility dose-volume histogram for rectum in Personalized template for a representative patient. The green, yellow, orange, and red regions in FDVH indicate that the goals are “achievable”, “challenging”, “difficult”, and “not achievable”, respectively. In this example, three dose-volume objectives were defined on the f = 0.1 curve (the black arrows) and one objective was set for the mean dose (the white circle).

An example of the Feasibility output window for a rectum volume is shown in **Figure 1**. The planner may then set new objectives for this OAR in terms of mean dose (the small circle) and/or dose-volume objectives (the arrows) before starting the optimization process.

In this study, the Pers plans were optimized using the *a-priori* fDVH knowledge for the main OARs supplied by the Feasibility module. In particular, the requested objectives for dose sparing were set on the f = 0.1 curve of the DVH (or mean dose panel) for each OAR (i.e. on the interface between the “challenging to achieve” and the “difficult to achieve” regions). In our experience, this choice provides provided the “optimal push” to the OAR goals without compromising target coverage.

**Figure 2:**
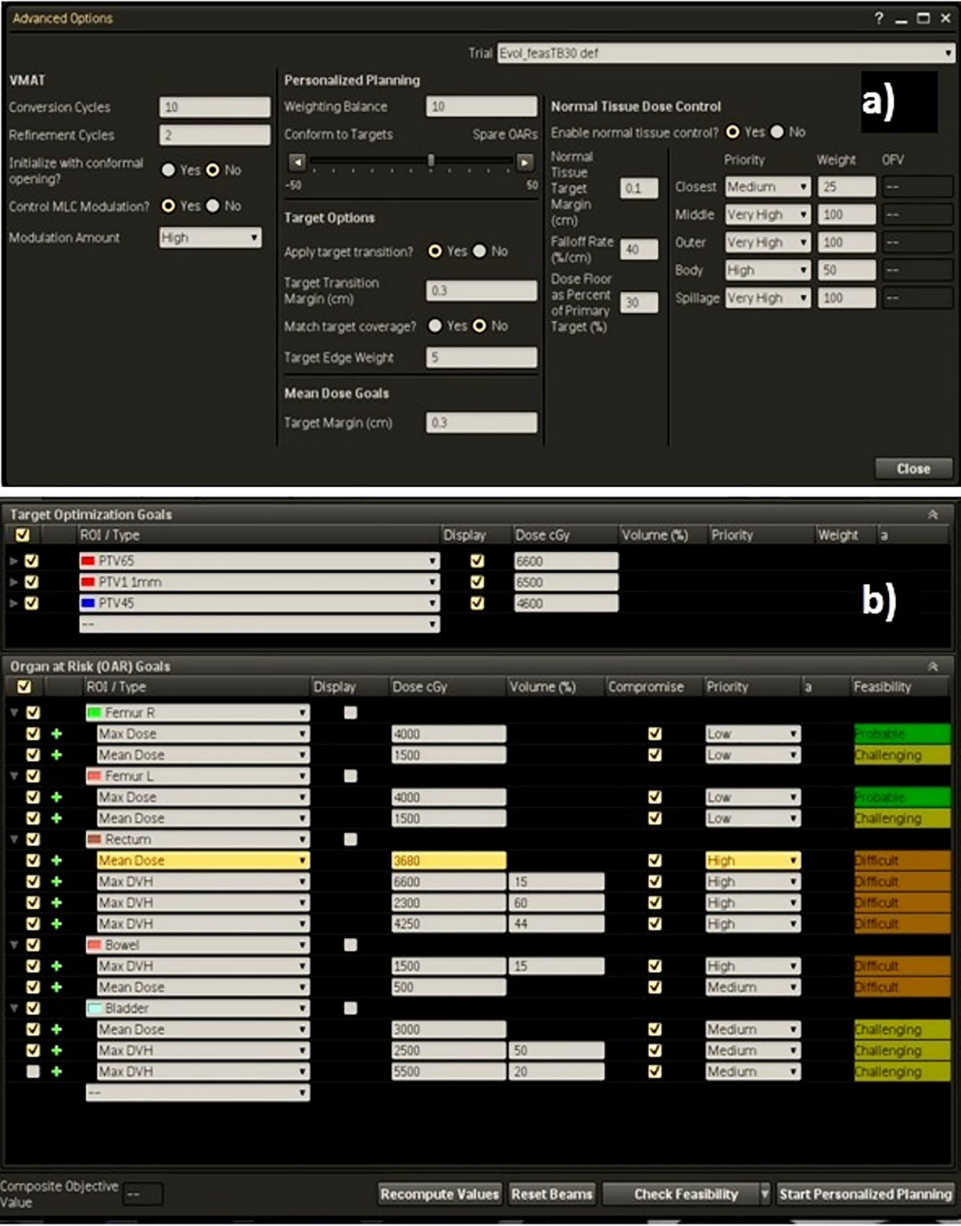
**(A)** Advanced settings template and **(B)** dose objectives for PTVs and OARs for a high-risk case.

As example, the Technique adopted for the high-risk prostate cancer patients was reported in **Figure 2**, showing (a) the template for advanced options and (b) the dose objective values used for optimization following the suggestions of the Feasibility module.

In this study, all automated AP and Pers plans were obtained in a single automated optimization round and had no manual intervention after the optimization process.

At the beginning of the implementation of our automated planning strategy, five patients for each anatomical site, not included in the present series, were used to create and tweak the initial Techniques in order to generate plans fulfilling the clinical objectives.

### Plan Evaluation

DVH analysis was used for plan comparison. The target volumes coverage were compared in terms of mean doses, D98%, D95%, and D2% (the doses to 98, 95, and 2% of target volumes). OARs dose sparing was evaluated following the metrics reported in **Table 1**.

For each PTV, a homogeneity index (HI) was calculated as:

$$HI=\frac{\left(D2\%-D98\%\right)}{D_{p}}$$

where Dp is the prescription dose. Closer the HI is to 0, better is the dose homogeneity.

The dose conformity (CN) was calculated for each target volume as suggested by the Van’t Riet et al. (32).

$$CN=\frac{TV_{RI}}{TV}\cdot \frac{TV_{RI}}{V_{RI}}$$

where TV_RI_ was the target volume covered by the reference isodose, TV was the target volume, and V_RI_ was the volume of the reference isodose. The first part of this equation defines the quality of target coverage and the second part defines the volume of healthy tissues receiving a dose greater than or equal to the prescribed dose. CN ranges from 0 (complete PTV geographic miss) to the ideal value 1 (perfect conformity of the reference isodose to the PTV). Reference isodose was selected as 95% of the prescribed dose.

Last, the integral dose (ID) received by non-tumor tissues was calculated as the product between mean dose and non-tumor tissue volume (Gy ∙ cc).

### Rectal NTCP Evaluation

The rectal normal tissue complication probabilities (NTCPs) were calculated for all patients using the Lyman-Kutcher-Burman (LKB) model (32). This model is based on a probit function:

$$NTCP=\frac{1}{\sqrt{2\pi }}\int_{-\infty }^{t(D,V)}\exp\left(\frac{-{\text{u}}^{2}}{2}\right)\text{du}$$

where

$$t=\frac{D-TD_{50}(V)}{m\cdot TD_{50}(V)}$$

$$TD_{50}(V)=\frac{TD_{50}(1)}{V^{n}}$$

where the parameters D, n, m, and TD_50_(1) determine the EUD delivered to the OAR of interest, the volume dependence of NTCP, the slope of NTCP *vs.* dose curve, and the tolerance dose to the whole organ leading to a 50% complication probability, respectively.

Specific complication endpoints for the rectum were selected as rectal bleeding of grade 1, 2, and ≥2. The corresponding set of parameters for TD50, n, and m are taken from literature (33–35) and reported in **Table 2**.

**Table 2:** Parameters used in calculation of normal tissue complication probability (NTCP) for rectal toxicity.

	n	m	TD50	Endpoint	Reference
NTCP1	0.14	0.26	59.2	Grade 1 rectal bleeding	Gulliford (34)
NTCP2	0.12	0.14	68.2	Grade 2 rectal bleeding	Gulliford (34)
NTCP3	1.00	0.16	55.9	≥ Grade 2 rectal bleeding	Tucker (35)

### Planning Efficiency

For each patient, the total number of monitor units (MUs), the treatment delivery time, and the total planning time (human inputs, optimization loops, and dose calculation times) were analyzed in order to evaluate the cost effectiveness of the automation procedure. All optimization processes were performed on a centralized server architecture (Oracle Pinnacle Professional X6-2, 22-core 2.20 GHz processor).

### Dose Delivery Verification

All plans underwent a detailed dosimetric verification in order to assess their deliverability accuracy. Delivered dose distributions were measured using a 2D ion-chamber array, the PTW 1500 Octavius detector, together with the Octavius-4D phantom both developed by PTW (PTW, Freiburg, Germany). This array consists of a matrix of 1,405 vented plane-parallel ion chambers of 4.4 mm × 4.4 mm × 3.0 mm in size, providing a maximum field size of 27 cm × 27 cm. This array is then inserted into the Octavius-4D motorized cylindrical polystyrene phantom. This phantom is capable to rotate synchronously with the gantry, in terms of angle and rotation speed, so that the detector array is always perpendicular to the beam then allowing the possibility of three-dimensional dose reconstruction. The measured dose distributions were then compared with the calculated ones using the gamma function concept. Following the suggestions of the AAPM report No. 218 (36), we considered the dose verification as optimal if the gamma index criteria exceeded 95% with 3%-2mm criteria for dose and distance-to-agreement.

### Statistical Analysis

A Kruskal-Wallis analysis of variance (ANOVA) was performed for statistical comparisons of data. The Bonferroni-Dunn *post-hoc* non-parametric test was run to correct for multiple comparisons, with p-values at 0.05 indicating statistical significance.

## Results

All manual and automated generated plans fulfilled the criteria for clinical acceptability in terms of OAR sparing and target coverage.

### Target Coverage

**Table 3** reports the dosimetric data for the PTVs. The PTVs coverage for MP, AP, and Pers plans is approximately equal for all parameters with no significant statistical differences. In particular, all plans in both risk groups achieved D95% ≥ 98% and D98% ≥ 95% for both PTVs. Automated AP and Pers plans resulted in a statistically significantly reduction of high-doses (D2%) in both cohorts, although the difference is small in absolute terms. The dose conformity was significantly better with AP and Pers plans than with MP plans in both scenarios, with Pers plans outperforming the AP plans and demonstrating a higher capability to better conform the doses to target volumes, especially to the complex concave lymph-nodal volumes. This was evident in the significant increased value of CN indexes.

**Table 3:** Comparison of dosimetric metrics between manual and automated plans for target volumes.

	Low-risk prostate							High-risk prostate						
	MP	AP	Pers	p	p			MP	AP	Pers	p	p		
				Kruskal-Wallis	MP *vs* AP	MP *vs* Pers	AP *vs* Pers				Kruskal-Wallis	MP *vs* AP	MP *vs* Pers	AP *vs* Pers
PTV65														
D98% (Gy)	62.4 ± 0.8	62.9 ± 0.9	62.7 ± 0.8	0.144	0.058	0.158	0.631	63.0 ± 0.6	63.2 ± 0.4	63.2 ± 0.3	0.212	0.112	0.146	0.895
D95% (Gy)	63.0 ± 0.8	63.7 ± 0.9	63.7 ± 0.8	0.616	0.394	0.394	0.986	63.4 ± 0.7	63.6 ± 0.4	63.7 ± 0.2	0.127	0.094	0.067	0.877
D2% (Gy)	68.1 ± 1.1	67.0 ± 1.0	67.5 ± 0.9	**0.029**	**0.017**	**0.026**	0.877	68.3 ± 0.8	67.4 ± 0.3	67.3 ± 0.7	**0.005**	**0.011**	**0.002**	0.612
Dmean (Gy)	65.6 ± 0.7	65.4 ± 0.9	65.8 ± 0.8	0.282	0.419	0.434	0.111	66.2 ± 0.7	66.3 ± 0.3	66.0 ± 0.4	0.310	0.310	0.628	0.134
H I	8.7 ± 2.0	6.3 ± 1.2	6.8. ± 1.5	**0.008**	**0.004**	**0.013**	0.692	8.2 ± 0.6	6.4 ± 0.9	6.2 ± 1.1	**<0.001**	**<0.001**	**<0.001**	0.986
PTV45														
D98% (Gy)	–	–	–	–	–	–	–	42.6 ± 0.3	43.1 ± 0.4	43.4 ± 0.4	**0.001**	**0.021**	**<0.001**	0.112
D95% (Gy)	–	–	–	–	–	–	–	43.5± 0.2	44.1 ± 0.4	44.3 ± 0.3	**0.001**	**0.002**	**<0.001**	0.300
D2% (Gy)	–	–	–	–	–	–	–	61.1 ± 2.5	58.8 ± 2.4	56.0 ± 2.7	**0.001**	0.096	**<0.001**	0.054
Dmean (Gy)	–	–	–	–	–	–	–	47.6 ± 1.6	45.9 ± 0.2	46.2 ± 0.4	**0.001**	**<0.001**	**0.001**	0.494
H I	–	–	–	–	–	–	–	41.0 ± 5.9	34.9 ± 5.6	28.0 ± 5.5	**0.001**	0.094	**0.001**	**0.027**
Dose conformity														
CN1	0.81 ± 0.05	0.86 ± 0.05	0.87 ± 0.05	**0.046**	**0.039**	**0.026**	0.877	0.80 ± 0.03	0.82 ± 0.03	0.83 ± 0.03	0.061	0.105	**0.021**	0.491
CN2	–	–	–	–	–	–	–	0.60 ± 0.04	0.67 ± 0.04	0.69 ± 0.02	**0.001**	**0.003**	**<0.001**	0.117

Bold values are the statistically significant ones.

**Figure 3:**
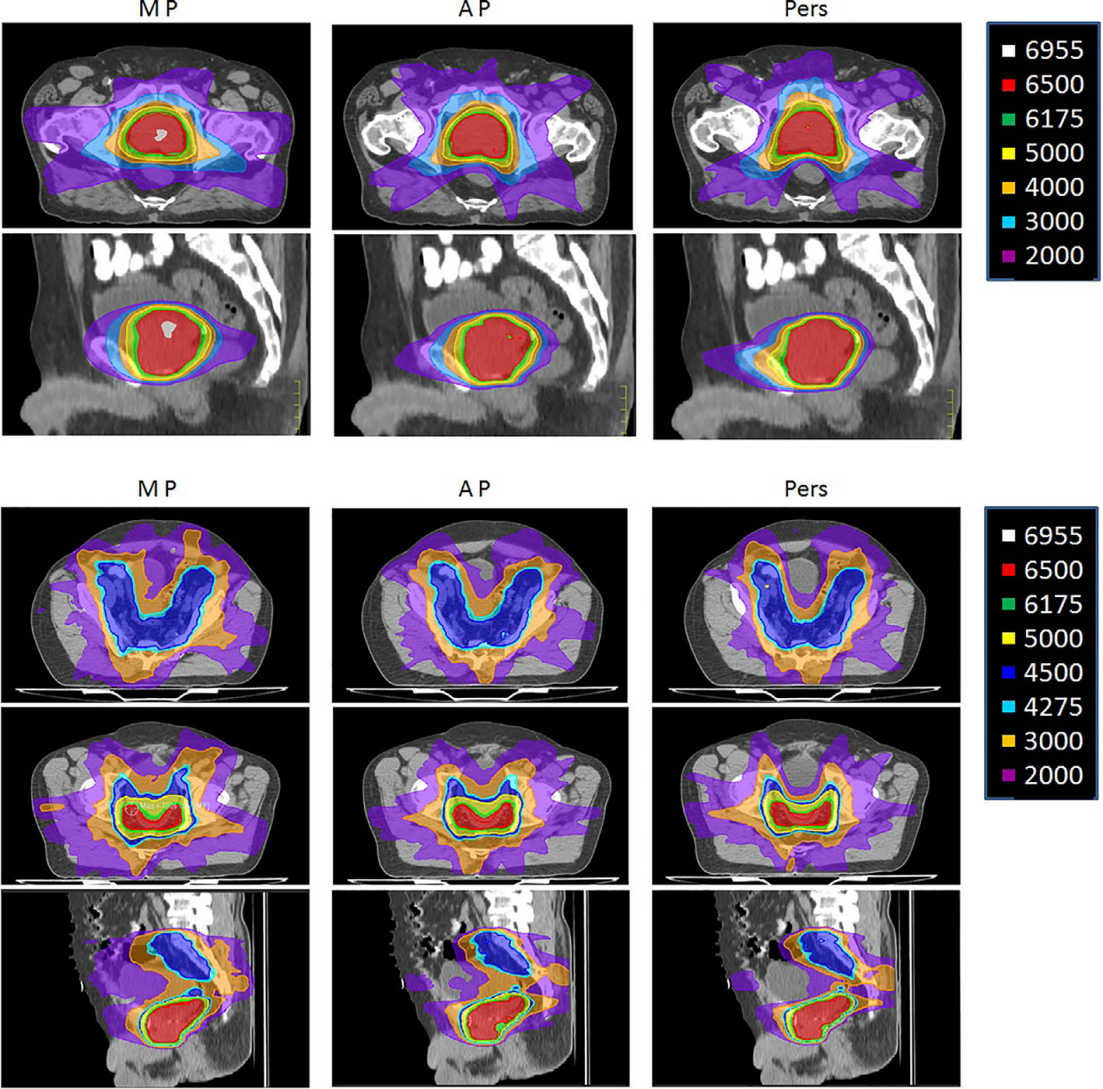
Representative dose distributions for manual plans (MP), Autoplanning (AP), and Personalized plans (Pers) for (upper) low-risk and (lower) a high-risk prostate cancer patients.

**Figure 3** shows the isodose distributions for MP, AP, and Pers plans for two representative patients with low-risk and high-risk prostate cancer in axial, sagittal, and coronal planes.

### OARs Sparing

**Table 4** reports the dosimetric data for the OARs sparing. Significantly lower rectal and bladder doses were observed in automated AP and Pers plans with respect to MP plans, with Pers plans reporting the lower values. For rectum, Pers plans yielded an average mean dose lower by 32% (11.3 Gy) and 21% (8.3 Gy) with respect to MP plans for low-risk and high-risk cohorts, respectively. With respect to AP plans, Pers plans decreased the rectal mean dose by 8% (2.0 Gy) and 9% (3.1 Gy) for low-risk and high-risk cohorts, respectively. Similarly, the Pers plans decreased the bladder mean doses by 24% (7.3 Gy) and 17% (7.6 Gy) with respect to MP plans for low-risk and high-risk cohorts, respectively. Although no statistical significance, Pers plans reported also a decrease of 10% (4.1 Gy) with respect to AP plans in the high-risk cohort. For both rectum and bladder, no statistical differences were found in the dose range ≥60.1 Gy (i.e. ≥65Gy, EQD2) with respect to MP plans.

**Table 4:** Comparison of dosimetric metrics between manual and automated plans for OARs.

	Low-risk prostate							High-risk prostate						
	MP	AP	Pers	p	p			MP	AP	Pers	p	p		
				Kruskal-Wallis	MP *vs* AP	MP *vs* Pers	AP *vs* Pers				Kruskal-Wallis	MP *vs* AP	MP *vs* Pers	AP *vs* Pers
*Rectum*														
Dmean (Gy)	35.1 ± 5.8	25.8 ± 5.5	23.8 ± 5.6	**<0.001**	**0.002**	**<0.001**	0.429	40.0 ± 2.4	34.8 ± 2.1	31.7 ± 2.5	**<0.001**	**0.001**	**<0.001**	**0.032**
V50 (%)	26.9 ± 9.6	18.6 ± 8.6	17.9 ± 8.5	**0.026**	**0.030**	**0.014**	0.770	30.9 ± 6.5	26.3 ± 6.5	23.7 ± 6.4	**0.009**	**0.046**	**0.002**	0.298
V60 (%)	17.8 ± 6.8	14.2 ± 6.7	13.4 ± 6.4	0.080	0.079	**0.037**	0.737	22.8 ± 5.5	19.4 ± 5.1	17.7 ± 5.5	**0.010**	**0.048**	**0.003**	0.318
V65 (%)	13.3 ± 6.2	11.8 ± 6.2	10.9 ± 5.6	0.484	0.439	0.235	0.681	17.0 ± 4.9	14.9 ± 4.1	14.4 ± 4.1	0.268	0.197	0.134	0.836
V70 (%)	6.8 ± 2.8	6.6 ± 3.0	6.7 ± 3.0	0.971	0.857	0.959	0.816	10.4 ± 3.3	9.9 ± 3.1	9.9 ± 3.2	0.837	0.594	0.618	0.973
NTCP1 (%)	31.5 ± 3.4	25.1 ± 6.4	24.7 ± 7.1	**0.010**	**0.009**	**0.008**	0.945	33.3 ± 4.2	31.2 ± 4.9	29.8 ± 5.2	**0.018**	0.078	**0.005**	0.286
NTCP2 (%)	6.0 ± 1.7	3.8 ± 2.3	3.7 ± 2.4	**0.016**	**0.018**	**0.010**	0.823	7.3 ± 1.7	6.3 ± 1.6	5.8 ± 1.5	**0.011**	**0.043**	**0.003**	0.362
NTCP3 (%)	1.9 ± 1.4	0.5 ± 1.4	0.2 ± 0.6	**<0.001**	**<0.001**	**<0.001**	0.925	3.3 ± 1.3	1.2 ± 0.5	0.5 ± 0.4	**<0.001**	**0.001**	**<0.001**	0.029
*Bladder*														
Dmean (Gy)	30.9 ± 9.9	23.7 ± 8.7	23.6 ± 8.7	0.054	**0.042**	**0.032**	0.904	44.2 ± 5.9	40.7 ± 6.4	36.6 ± 6.8	**0.014**	0.146	**0.004**	0.146
V65 (%)	16.7 ± 8.2	14.8 ± 8.2	15.9 ± 8.1	0.632	0.348	0.763	0.524	20.3 ± 12.6	19.7 ± 11.6	18.8 ± 12.3	0.798	0.817	0.508	0.667
V70 (%)	12.6 ± 5.5	12.0 ± 7.5	12.1 ± 7.0	0.616	0.380	0.409	0.959	16.0 ± 10.7	15.9 ± 9.9	15.9 ± 10.9	0.946	0.938	0.808	0.749
*Femoral head R*														
Dmean (Gy)	14.5 ± 3.7	12.3 ± 4.5	9.3 ± 2.5	**0.001**	0.089	**0.001**	0.056	22.8 ± 4.5	20.9 ± 2.1	19.7 ± 2.4	**0.041**	0.204	**0.011**	0.204
V45 (%)	3.1 ± 5.3	0 ± 0.0	0 ± 0.0	**<0.001**	**<0.001**	**<0.001**	1.000	0.3 ± 0.4	0 ± 0.0	0 ± 0.0	**<0.001**	**<0.001**	**<0.001**	1.000
*Femoral head L*														
Dmean (Gy)	14.2± 3.4	11.7± 4.4	9.5± 2.8	**0.012**	0.130	**0.003**	0.144	22.3 ± 2.4	20.6 ± 2.9	19.9 ± 2.2	**0.049**	0.148	**0.015**	0.322
V45 (%)	1.9 ± 3.2	0 ± 0.0	0 ± 0.0	**<0.001**	**<0.001**	**<0.001**	1.000	0.5 ± 0.8	0 ± 0.0	0 ± 0.0	**<0.001**	**<0.001**	**<0.001**	1.000
*Small Bowel*														
Dmean (Gy)	–	–	–					12.9 ± 4.9	12.3± 4.3	11.9 ± 4.4	0.859	0.895	0.597	0.691
V15 (cc)	–	–	–					115.1 ± 44.5	115.4 ± 47.8	113.1 ± 45.8	0.953	0.774	0.808	0.965
*Healthy tissues*														
ID (Gy*cc*10^5^)	1.38 ± 0.31	1.17 ± 0.29	1.16 ± 0.28	0.135	0.095	0.074	0.904	2.63 ± 0.36	2.44 ± 0.35	2.34 ± 0.33	0.139	0.291	0.057	0.354
V30 (cc)	707 ± 204	679 ± 232	659 ± 231	0.775	0.712	0.475	0.731	2,600 ± 282	2,099 ± 306	1,842 ± 272	**<0.001**	**0.008**	**<0.001**	0.107
V10 (cc)	4,363 ± 1,042	4,293 ± 1,032	4,140 ± 1,026	0.799	0.891	0.525	0.618	9,318 ± 1,379	9,232 ± 1,488	8,974 ± 1,386	0.759	0.877	0.481	0.581

Bold values are the statistically significant ones.

Doses to the femoral heads were significantly lower in Pers plans, with mean dose reductions of about 5 and 3 Gy with respect to MP plans and 2 and 1 Gy with respect to AP plans for low-risk and high-risk patients, respectively. In the high-risk cohort, no significant dose differences were found for the small bowel irradiation among the three planning techniques.

The integral dose was found significantly lower with the automated plans, with a reduction of ID of about 11–16% for the Pers plans and 7–15% for the AP plans, with respect to MP plans. In general, larger volumes of normal tissues were exposed to low doses in AP plans, particularly at doses ≤30 Gy, as reported in **Table 4**. For example, the volume of the 30 Gy isodose was decreased on average by 48 and 758 ml in the two patient cohorts with Pers plans compared to MP plans, and by 20 and 257 ml compared to AP plans. This feature can also be seen in the dose distributions shown in **Figures 2A, B** for two representative patients of the two cohorts.

**Figure 4:**
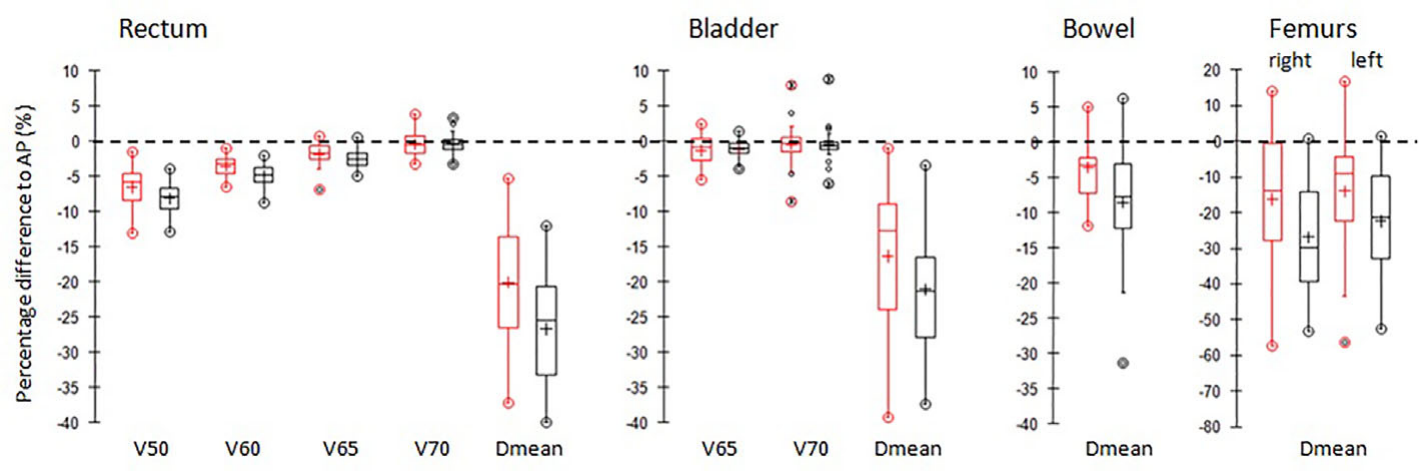
Boxplots of differences of the main dosimetric metrics of AP plans (red) and Pers plans (black) compared to MP plans for rectum, bladder, femurs, and small bowel irradiation. PTVs. The central line marks the median, the edges of the box are the 25th and 75th percentiles, black circles represent the extreme values. The crosses represent the mean values.

**Figure 4** shows the box-plots of relative percentage differences in dosimetric parameters for the main OARs of AP (black) and Pers (red) plans with respect to MP plans for all patients.

**Figure 5:**
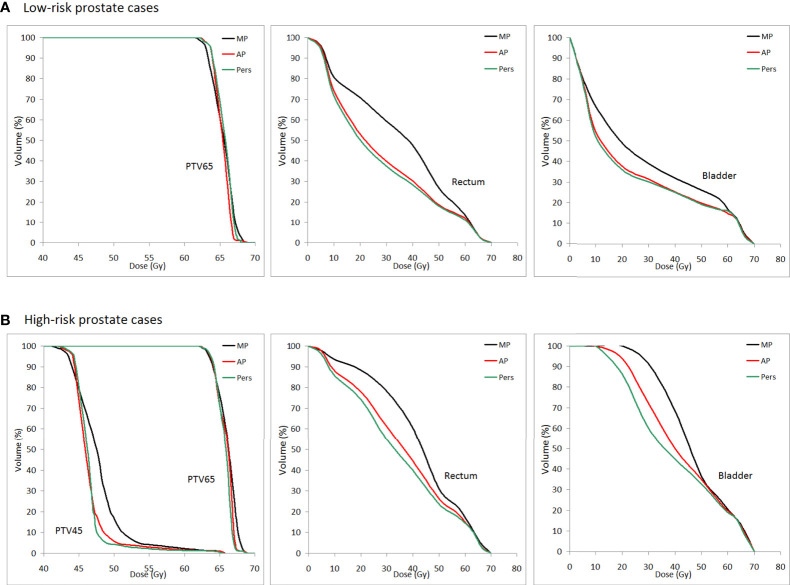
Population mean DVHs for PTV65, PTV45, rectum, and bladder for **(A)** low-risk and **(B)** high-risk prostate cancer patients (MP plans: black-solid line, AP plans: red solid-line, and Pers plans: green solid-line).

**Figure 5** shows the average DVH curves of the plans for (a) low-risk and (b) high-risk cancer cases.

### Rectal NTCP Evaluation

***T***he calculated rectal NTCPs for all patients are reported in **Table 4**. With respect to grade ≥2 rectal bleeding toxicity (NTCP3), AP and Pers plans resulted in a significantly lower NTCP with respect to MP plans. In this case, the n value corresponds to a parallel tissue architecture, therefore the gains in rectal DVH with automated plans are expected to be due to the major decrease of the rectal mean dose. Interestingly, since the parameter set NTCP1 and NTCP2 have an n value corresponding to more serial tissue architecture, the decrease of NTCP observed with automated plans corresponds to a dose decrease in the high-dose region, although of lesser amount.

### Planning Efficiency and Dose Verification

The average MU number, the total planning time, the delivery time, and the results for dose delivery verification are given in **Table 5**. Averaged over all patients, Pers plans required about 14 and 76% more MUs than AP and MP plans for low-risk prostate and about 20 and 39% more MUs than AP and MP plans for high-risk prostate cancer patients, respectively. Despite the large differences in MUs, the total “beam-on” times for Pers plans were only about 1 min longer than MP plans and similar to those of AP plans.

**Table 5:** Overview of planning efficiency and treatment delivery metrics.

	Low-risk prostate							High-risk prostate						
	MP	AP	Pers	p	p			MP	AP	Pers	p	p		
				Kruskal-Wallis	MP *vs* AP	MP *vs* Pers	AP *vs* Pers				Kruskal-Wallis	MP *vs* AP	MP *vs* Pers	AP *vs* Pers
MUs	374 ± 51	578 ± 79	657 ± 81	**<0.001**	**<0.001**	**<0.001**	0.113	528 ± 58	613 ± 53	736 ± 63	**<0.001**	**0.029**	**<0.001**	**0.014**
Planning time (min)	63.0 ± 15.5	15.8 ± 0.9	6.7 ± 0.2	**<0.001**	**<0.001**	**<0.001**	**<0.001**	138.5 ± 48.1	60.6 ± 4.1	15.0 ± 0.6	**<0.001**	**<0.001**	**<0.001**	**<0.001**
Beam-on time (min)	1.2 ± 0.2	1.9 ± 0.3	2.1 ± 0.3	**<0.001**	**<0.001**	**<0.001**	0.185	2.0 ± 0.2	2.4 ± 0.2	2.7 ± 0.3	**<0.001**	**0.009**	**<0.001**	**0.024**
γ pass-rate (%)	98.7 ± 1.2	98.1 ± 1.4	98.0 ± 1.4	0.368	0.281	0.184	0.802	97.7 ± 1.2	97.2 ± 1.6	96.6 ± 1.7	0.238	0.403	0.147	0.252

Bold values are the statistically significant ones.

The average treatment planning time was found to decrease dramatically in the transition from manual to automated planning. Using a centralized server architecture, Pers plans were generated with average times of about 7 and 15 min for low-risk and high-risk patient cohorts, respectively. These planning times were significantly lower also compared with AP planning times, with an average decrease of about 9 and 45 min for the low-risk and high-risk cohorts, respectively.

Pre-treatment verification was performed for all plans. With criteria equal to 3% (global) −2 mm for γ-index, the average pass-rate was greater than 95% for all plans and all techniques.

## Discussion

The advancement in artificial intelligence is reshaping the field of radiation oncology in all aspects. In particular, the continued evolution of computerized solution for automated treatment planning are advancing physicists’ ability to generate high-quality treatment plans. With regard to low-risk prostate cases, Heijmen et al. (36) presented a summary of previously published studies between automated and manual generated plans. Most of these studies were focused on the knowledge-based strategy (12–15) reporting only small differences in dosimetric endpoints. On the contrary, in their multi-center study (37), the Erasmus-ICycle engine was used for MCO-based automation of prostate cancer planning in four Centers, reporting an overall dosimetric superiority of automated plans in terms of rectal dose reduction. The same research group used the ICycle engine to explore the patient-specific trade-offs between planning aims in prostate cancer (38). The authors reported significant NTCP reductions for rectal toxicity and underlined the role of automated approach for personalization of patient care. Studies for high-risk prostate cancer or for complex pelvis treatment are much rarer. In a recent review focused on automated planning, Hussein et al. (39) identified only one out of the 81 studies on whole pelvic prostate radiotherapy. This study, performed by Buschmann et al. (19), evaluated the Erasmus-ICycle planning automation solution as a pre-optimizer for automated VMAT planning. Automated VMAT plans exhibited strongly improved organ sparing and higher conformity compared to manual plans, with mean doses of bladder and rectum reduced by 10.7 and 4.5 Gy, respectively. Recently, we evaluated the potential of Pinnacle Autoplanning for head-neck, endometrial, and high-risk prostate cases, reporting a significant increase of dose conformity and a reduction of plans variability and planning times (21). To the authors’ knowledge only another study has been published in this clinical setting. Wheeler et al. (40) evaluated a novel automated planning solution whose Pareto navigation-based methodology enabled clinical decision-making on trade-off balancing to be incorporated within automated protocols. The authors successfully applied their engine to prostate cancer patients with and without elective nodal irradiation and robustly generated high quality plans in an efficient manner.

Starting from this limited evidence base, our study provided further data in support of automation for two prostate treatment scenarios of different complexity. In particular, we evaluated for the first time the potential of a new fully automated template-based VMAT planning engine, called Pinnacle Personalized, in low-risk and high-risk prostate cancer patients. In the last scenario, treatment involves large concave-shaped targets and multiple dose prescriptions, and therefore presents a major challenge for the automated engines algorithms. In the present study, no differences were observed for target coverage, but AP and Pers automated plans reported an overall improvement of plan quality in terms of dose conformity and sparing of critical structures, with Pers plans outperforming also the AP plans. In addition to the availment of the templates that is common with the Autoplanning optimization procedure, the driving force of the new Personalized automated planning engine was found in its integration with the Feasibility module. The use of the Feasibility engine translated in a significant reduction of rectal dose not only compared to manually generated plans but also with respect to AP plans. The average mean dose to rectum was decreased by 32.2 and 7.8% in the low-risk scenario and by 20.8 and 8.9% for the high-risk cases with respect to MP and AP plans, respectively. Most of these dose reductions are in the low and middle dose range. In other words, we reported that an “*a priori*” knowledge of the theoretical dose-volume space available for each OAR had a substantial impact on plan quality, able to identify for each patient dosimetric outliers and planning cut-off criteria. This feature is a major step forward not only with respect to conventional manual planning but also with respect to Autoplanning strategy. In conventional manual planning, due to lack of knowledge of achievable dose sparing for a particular anatomy, the planner does not exactly know when to adjust, where to adjust, and even when to stop optimization. This means that even an experienced planner does not know whether an optimal plan has been achieved without clear knowledge on the correlation between anatomy features and achievable DVH. Then, based on its own experience and skills, a planner has to rely on the additional adjustments till no further improvement can be achieved. For example, in the present study, the evaluation of manual generated plans showed that the doses to normal structures were far below the institutional objective constraints, then all MP plans were considered optimally generated and clinically acceptable and no further optimizations were performed.

The price to pay for this quality improvement was an increase in the plan complexity. Automated AP and Pers plans were found associated with a large number of monitor units and small and complex control points. The increase of MUs number may lead to more head scatter and higher peripheral doses, potentially contributing to an increase of total body radiation dose. However, unlike expected, the increase of MUs number did not increase the integral dose to the patients; mean ID was found lower by 16 and 11% for the Pers plans with respect to MP plans, theoretically reducing the risk of secondary malignancies (41). In addition, this increased plan complexity might also lead to challenges in radiation delivery, since higher plan complexity has been associated to inaccurate dose delivery and worse quality assurance outcomes (42). In order to better understand the trade-off between plan complexity and the dosimetric accuracy of the treatment delivery, we performed a “pre-treatment” dose verification of all plans. Despite the higher complexity of automated plans, the results of dosimetric verification confirmed the deliverability of the AP and Pers plans and their reliability for clinical applications. Another shortcoming of the MUs increase is the prolongation of about 1 min of the beam-on time for automated plans. If this extra-time may theoretically have an impact on intra-fraction prostate motion, it has been recently reported (43) that applied target margins as those used in this study are adequate to mitigate intra-fraction motion of the prostate for total treatment durations up to 8 min.

A major finding supplied by the new Personalized engine is the impressive reduction of planning time. The mean overall time, including human inputs, optimization loop processes, and calculation times, was less than 7 min for low-risk prostate and 15 min for high-risk prostate cases, respectively. The dramatic reduction of planning times can open up new possibilities for a real-time adaptive radiotherapy. The precise targeting of the prostate and the pelvic lymph nodes is challenging because both targets move independently, with shift up to 15 mm day to day (44). Then the intra- and inter-fraction motion of the prostate may negate the advantages of highly conformal dose distributions obtained by VMAT. In particular, since the prostate is highly mobile (due to differences in bladder and rectum filling) while the pelvic lymph nodes are less mobile (due to their close proximity to vascular structures) a simple correction of the isocenter position to compensate for prostate motion may reduce the pelvic lymph nodes dose coverage, particularly in highly modulated treatments. Therefore, daily inter- and intra-fraction anatomical changes need to be accounted for both targets at the same time. Adaptive radiotherapy (ART) has been proposed to either reduce or compensate for the effect of patient-specific treatment variation measured during the course of radiotherapy using offline adaptive re-planning (45) of pre-planned libraries (46). Recently, the introduction of MR linacs offers new possibilities for daily adaptive re-planning in prostate cancer, thanks to high soft tissue contrast imaging (47). However, all these approaches are hampered by the time-consuming re-planning process, representing nowadays the major obstacle for large scale implementation of ART strategy. The major improvement of planning efficiency supplied by the Personalized engine has the potential to make routine online adaptive radiotherapy a possibility, allowing prostate cancer patients to be treated with a plan adapted according to actual anatomy in a few minutes after imaging. These new opportunities are in some way a response to the alarms raised about the impact that AI may have on the current organization of medical physics and dosimetry departments; in particular, the question if AI technology will marginalize medical physicists in the near future has been recently debated (48). As fairly expressed by Moore et al. (49), if this new technology is able to increase our ability to plan faster and more frequently as promised by the adaptive radiotherapy concept, then the positions of dosimetrists and medical physicists *“may be used for dose aggregation, analysis, individualized care, and many other activities which were not possible with conventional clinical practices.”* In other words, in our opinion, the demand for clinical medical physicists can only increase as technologies such as AI are becoming more complex in healthcare. A current research is ongoing in our Center to expand the Personalized engine planning in an integrated workflow with online adaptation to be able to generate new plans on demand.

A direct comparison between knowledge-based and template-based algorithms was performed for head-neck tumors in order to assess the strengths and/or weaknesses of the two automation strategies, reporting comparable results (50). However, compared to these alternative methods for automation of treatment planning, the Autoplanning and Personalized engines present a clear alternative. For knowledge-based systems, a library of prior patients is required to build up the corresponding mathematical model. This library must be filled with a large number of high-quality plans for each protocol and disease site, whose clinical implementation translated in a labor-intensive process. Any changes in contouring protocol or dose prescription or planning techniques could require the generation of a new database. Moreover, the newly generated plan quality inevitably depends on the quality of the plans building the database, so that non-optimal plans entered in the database may degrade results. On contrary, the Personalized (and Autoplanning) plan solutions are therefore not influenced by the quality or quantity of historical plans and new techniques can be easily developed without time consuming. In our experience only a small set of training patients for each anatomical site (five patients) was necessary as starting point for the implementation of the Techniques in both Autoplanning and Personalized engines by an expert team of medical physicists and radiation oncologists. Moreover, also the Feasibility module does not require a database of prior plans but rather derives the lower achievable boundary of the dose volume histograms for the OARs from nearly first principles, only assuming that the targets are uniformly covered with the prescription doses. The Feasibility solutions should then be Pareto optimal, i.e. one or more objectives (as OARs sparing) cannot be improved without worsening at least one other (as target coverage). However, this demonstration is a challenging mathematical task and is beyond the scope of the present paper.

Furthermore, AP algorithms may provide easier access to complex and high-quality radiotherapy treatments, improving the consistency between treatments carried out in different institutions. In fact, for each anatomical site, it is possible to define a standardized model which can then be shared and adapted to the local practice of many different centers. Therefore the diffusion of AP model configurations represents a solid strategy for the dissemination of optimized plans (22).

Lastly, some limits need to be recognized. First, the validation of a model for clinical use requires important skills and huge background knowledge of the medical physicists that has to wisely balance the trade-offs between the sparing of OARs and targets coverage. If the model would result in suboptimal implementation this would bias all treatments for that anatomic site. Secondly, the impact of quality of manually generated plans has to be recognized, since the poorer the manual plan, the better the AP plans. For this reason all manual plans were optimized by a senior medical physicist with long-lasting VMAT planning experience whose endpoint was to achieve high-quality manual plans avoiding inter-planner variability. Last, this is a single institution study, therefore findings could be biased by local planning procedures and may not automatically translate in other centers with different equipment, procedures, protocols, and planning experience. The present study highlighted the potential of Pinnacle Personalized engine for prostate cancer treatments; currently, we are planning a multi-center study aimed to validate this new algorithm in other anatomical districts.

## Conclusion

Automation in treatment planning is a rapidly developing field and the new algorithms for plan optimization demonstrated the potential to increase the plans overall quality. We evaluated the Pinnacle Personalized engine to be a robust clinical tool, reporting significant increase of dose conformity with respect to manual planning and Autoplanning solutions in two different prostate treatment scenarios. The use of Feasibility module allows to push the limits of OAR sparing while maintaining routine clinical target coverage goals. Moreover, Personalized offers a dramatic reduction in planning times with the potential to make routine online adaptive radiotherapy a real possibility.

## Data Availability Statement 

The raw data supporting the conclusions of this article will be made available by the authors, without undue reservation.

## Ethics Statement 

Ethical review and approval was not required for the study on human participants in accordance with the local legislation and institutional requirements. Written informed consent for participation was not required for this study in accordance with the national legislation and the institutional requirements.

## Author Contributions 

SC, FD, and GM contributed to the study design. CR and VM contributed to planning and data collection. MB, ND, and LI participated in data analysis and interpretation. SC and FD contributed to writing the manuscript. AM and VV contributed to supervision and study management. All authors contributed to the article and approved the submitted version.

## Conflict of Interest

The authors declare that the research was conducted in the absence of any commercial or financial relationships that could be construed as a potential conflict of interest.
